# The Significance of Circulating Tumor Cells in Patients with Hepatocellular Carcinoma: Real-Time Monitoring and Moving Targets for Cancer Therapy

**DOI:** 10.3390/cancers12071734

**Published:** 2020-06-29

**Authors:** Feiyu Chen, Zhangfeng Zhong, Hor-Yue Tan, Ning Wang, Yibin Feng

**Affiliations:** School of Chinese Medicine, Li Ka Shing Faculty of Medicine, The University of Hong Kong, Hong Kong SAR 852, China; fychen@hku.hk (F.C.); zfzhong@hku.hk (Z.Z.); hyhtan@hku.hk (H.-Y.T.); ckwang@hku.hk (N.W.)

**Keywords:** circulating tumor cells, hepatocellular carcinoma, EMT, metastasis, cancer stem cells

## Abstract

Hepatocellular carcinoma (HCC) is ranked as the sixth most common cancer around the world. With the emergence of the state-of-the-art modalities lately, such as liver transplantation, image-guided ablation, and chemoembolization, the death rate is still high due to high metastasis rate after therapy. Observation by biannual ultrasonography allows effective diagnosis at an early stage for candidates with no extrahepatic metastasis, but its effectiveness still remains unsatisfactory. Developing a new test with improved effectiveness and specificity is urgently needed for HCC diagnosis, especially for patients after first line therapy. Circulating tumor cells (CTCs) are a small sub-population of tumor cells in human peripheral blood, they release from the primary tumor and invade into the blood circulatory system, thereby residing into the distal tissues and survive. As CTCs have specific and aggressive properties, they can evade from immune defenses, induce gene alterations, and modulate signal transductions. Ultimately, CTCs can manipulate tumor behaviors and patient reactions to anti-tumor treatment. Given the fact that in HCC blood is present around the immediate vicinity of the tumor, which allows thousands of CTCs to release into the blood circulation daily, so CTCs are considered to be the main cause for HCC occurrence, and are also a pivotal factor for HCC prognosis. In this review, we highlight the characteristics and enrichment strategies of CTCs, and focus on the use of CTCs for tumor evaluation and management in patients with HCC.

## 1. Introduction

Although there are improvements in the treatment strategy and patient stratification, hepatocellular carcinoma (HCC) still contributes to a significant burden of disease for both patients and health services. The global age-standardized incidence has increased to 10.1 cases per 100,000 person-years since 2012 [1]. Over 700,000 cases are diagnosed per annum [2], which are more common in men than women, as the main risk factors of hepatitis B virus (HBV), hepatitis C virus, and excessive alcohol intake are more prevalent and possibly more carcinogenic in males [3]. To date, generalized decisions for HCC treatment options are mainly based upon the traditional pathological characteristics of the primary tumor. With the emergence of the state-of-the-art modalities lately, such as liver transplantation, image-guided ablation, and chemoembolization, the death rate is still high owing to high metastasis rate after therapy [4]. Besides, patients with early-stage HCC have a well-preserved liver function and are free of symptoms [5]. Currently, surveillance by biannual ultrasonography is the recommended method, and this allows effective diagnosis at an early stage for candidates with no extrahepatic metastasis, but its effectiveness still remains unsatisfactory [6]. Therefore, developing a new test with improved effectiveness and specificity is urgently needed for tumor diagnosis, especially for patients after first line therapy. Notably, the current recommendation is shifting from the empirical strategies to the classification of the molecular profile of the tumor. Through sorting the molecular profiles and de novo mutations within a heterogenous population of HCC cells, specific tumor molecular and genetic data of each patient are identified. These improved methods facilitate the identification of low-frequency cells in HCC that may account for the heterogeneity involved in HCC presentation and responsiveness to the treatment [7,8].

Highly sensitive liquid biopsy assays have been developed to detect and analyze cells or organelles that are released from the tumor and enter into the blood circulatory system, such as circulating tumor cells (CTCs), circulating tumor DNA (ctDNA), and tumor-derived exosomes [9]. They have migrated into the blood circulation, but have not arrived to the next affected peripheral site, so these cells were regarded as the precursor “seeds and soil” of discrete tumors [10]. The molecular and genetic characteristics of these cells or organelles should ideally provide dynamic assessments of tumor behaviors and information on prognosis, metastasis, and possible drug resistance to drug treatment. This information potentially predicts accurate patient risk stratification and allow a timely transition of therapy selection, which allow the optimization of personalized therapeutic treatment and reduction in patient morbidity from unnecessary treatments [11].

In the 1860s, CTCs were identified for the first time using a microscope by Thomas Ashworth in the blood of a man with metastatic cancer [2]. Over the past 10 years, CTCs have been recognized as seminal biomarkers and attracted enormous attention. CTCs have also been used for prognosis in patients at an early stage or with metastatic disease to identify suitable adjuvant therapy or surveillance, and to act as a substitute biomarker during therapy [10]. Moreover, another crucial use for CTC detection and characterization is to serve as a “liquid biopsy” representative of the tumor. With the development of detection technologies, CTC profiles and molecular landscape can be obtained, and this provides vital information on the molecular characteristics of these cells in the blood circulatory system, as well as the inter- and intra-tumor heterogeneity [10].

In HCC, blood is present around the immediate vicinity of the tumor, and this allows thousands of CTCs to be released into the blood circulation daily. CTCs carry a lot of tumor information to invade into the blood, which is crucial for the metastatic cascade from a functional point of view [12]. Only a portion of cases can be diagnosed when multiple metastatic lesions are developed, which consequently misses the best time window for surgical resection. Upon invasion by CTCs into the blood vessels, their immediate detection at this early stage may serve as an efficient tool for tumor surveillance. Moreover, HCC displays a heterogeneous profile, and the phenotypes of CTCs in HCC change dynamically. In the course of cancer metastasis, a multi-step and multi-factorial process occurs, and the phenotype at one site could not represent the tumor characteristics [13]. Consequently, the phenotypic profiling of CTCs could be employed for real-time disease monitoring and prompt decisions for the therapeutic options [12].

**Figure 1:**
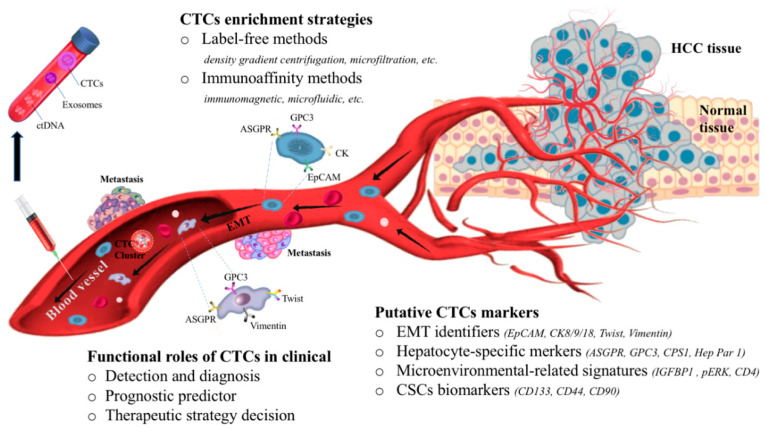
The biology and clinical potential of circulating tumor cells (CTCs) in hepatocellular carcinoma. CTCs enter into the bloodstream, most of the CTCs are destroyed by sheer stress, anoikis, or immune destruction, and only a few of them undergo the EMT (epithelial to mesenchymal transition) process. With the complexity of the microenvironment, CTC biomarkers of hepatocellular carcinoma (HCC) are divided into EMT-related (e.g., EpCAM, cytokeratin (CK), twist, and vimentin), hepatocyte-specific (e.g., asialoglycoprotein receptor (ASGPR), glypican-3 (GPC3)), microenvironmental-related (e.g., insulin like growth factor binding protein 1 (IGFBP1), phosphorylated ERK (pERK), CD4), and stem-like phenotypes (e.g., CD133, CD44, CD90). The techniques used to detect and collect CTCs are “label-free” and immunoaffinity methods, based on the biophysical and biochemical properties of CTCs, respectively. For the management of HCC, CTCs can provide diagnostic and prognostic information of HCC progression, facilitate accurate patient risk stratification, and allow a timely optimization of personalized therapeutic treatment.

CTCs were investigated in numerous clinical trials, in which their clinical utility remains a big challenge. There are many issues, including single-center study, relatively small cohort size, and short follow-up time, that limit its use clinically [14,15]. In this review, we present an overview of the capture technologies and current retrospective studies with clinical insights into the CTC heterogeneity in HCC. In particular, we address some open questions in this basic subject of CTCs, focus on the putative role of CTCs in monitoring HCC progression, and discuss their characteristic phenotypes in clinical studies (Table 1 and Figure 1). Our aim is to provide the current understanding of CTCs in HCC, so this can contribute to the translation of experimental data into clinical studies.

## 2. Overview of CTC Enrichment Strategies

CTCs refer to a small sub-population of tumor cells in human peripheral blood, which are released from the primary or metastatic tumor and invade into the blood or lymphatic circulatory system [43]. Most of the CTCs travel as individual cells, but some are as clusters, also called microemboli [44], which particularly result in advanced tumors [45]. There is evidence showing that in several types of cancer, CTCs in clusters are more potent than single CTCs in infiltrating into the distant tissues and forming metastases [10,46]. Besides, some CTCs can interact with platelets in the bloodstream [47], they rely on platelets in mediating and enhancing extravasation [48]. So how can we isolate CTCs when they are present in low levels and background components (e.g., erythrocytes, leukocytes) are co-isolated with them in the blood samples?

As early as the 1950s, Fawcett et al. were the first to demonstrate that density gradient centrifugation could be used for concentration and segregation of malignant cells, where tumor cells are separated under centrifugal forces from bloody, pleural, and peritoneal fluids [49]. However, this method was not widely used due to it being labor-intensive, time-consuming, and having poor efficiency. Recently, a variety of technologies were developed to enrich CTCs [50,51], most of them can be divided into two sub-categories based on the biophysical and biochemical properties of CTCs, which are usually referred as “label-free” and immunoaffinity methods, respectively. Label-free methods make use of the intrinsic contrast of the cell properties, such as density, size, deformability, and electric charge, to discriminate CTCs from erythrocytes and leukocytes [52]. Therefore, techniques such as microfiltration, hydrodynamic, acoustophoresis, and dielectrophoresis, were developed [52,53,54]. For examples, size-based filtration devices were developed based on different sizes of CTCs, and they utilize inertial forces to isolate CTCs and leukocytes into separate streamlines [55]. A device named Labyrinth combines size- and immunoaffinity-based approaches, and has been used in practice to isolate CTCs from the peripheral blood of the HCC patients [33]. 

CTCs enrichment based on their biophysical properties can be achieved through high-throughput and label-free enrichment, but it faces challenges in enhancing the specificity, as well as the purity of the enriched CTCs [53,54,56]. Unlike “label free” physical attribute-based methods, immunoaffinity-based methods rely on the highly specific interaction between ligands (antibodies or chemical antibodies) and tumor-specific antigens that are present on the cell membrane of CTCs [52]. The affinity ligands are immobilized either on a microdevice or magnetic beads with enhanced surface-to-volume ratio to achieve high capture efficiency with high purity [57,58]. In most of the immunoaffinity methods (e.g., immunomagnetic, microfluidic), epithelial cell adhesion molecule (EpCAM) is a representative biomarker, which is expressed on the cell surface of CTCs [59]. The CellSearch system, the only FDA (the US Food and Drug Administration)-approved technology for the enrichment of CTCs in metastatic cancer, isolates cells based on their EpCAM expression on the cell surface [52,60]. This method has been used in HCC patients to study the correlation of CTCs in the presence of EpCAM in patients, who survive after curative resection [17,40]. However, this technique may fail to isolate CTCs that do not express EpCAM [61], including the cells that have undergone a dynamic process of epithelial to mesenchymal transition (EMT), in which EpCAM expression is downregulated or even undetectable [62,63]. Lately, the CanPatrol system has become more popular in this field. It combines microfiltration and multiple RNA in situ hybridization for CTC identification and simultaneous classification, which is based on the antigens that are expressed on the cell surface of CTCs [19,20,21,23,24,25,26]. This method has been used in a range of carcinomas including HCC. For example, with the use of many other biomarkers, such as twist and vimentin, CTCs were more efficiently enriched from the blood samples of the HCC patients [19,20,21,23,24,25,26].

Collectively, the conceptual description and performance of CTC detection and capture technologies in the context of clinical practice has been reviewed in previous literature [52,64,65]. Therefore, this section only provides a general overview in this field, and the representative strategies described here serve as a starting point for audiences who are interested in the research of CTC enrichment technologies.

## 3. The Dynamic Changes of CTCs in Blood Circulation

Thousands of CTCs are generated each day, but most of the CTCs are destroyed by sheer stress, anoikis, or immune destruction [66]. In the blood circulation, the concentration of CTCs is extremely low, and its rarity is a big challenge for detection and characterization. To date, some capturing systems have been developed to detect this rare population from the body fluids [52,64,65]. Less than one CTC was detected in 10^6^ of the peripheral blood mononuclear cells in breast cancer [67], and around 1 mL of the blood comprised five CTCs in bladder cancer patients [68]. In fact, the number of CTCs is associated with patient prognosis. The clinical data have shown that patients with fewer than five CTCs per 7.5 mL of the peripheral blood had longer progression-free survival (PFS) and overall survival (OS) than those with equal to or higher than five per 7.5 mL of blood in several types of cancers including colorectal, breast, prostate, and non-small-cell lung cancers [69,70,71,72]. Although these intriguing observations were seen in a variety of cancers, little is known on the persistence of CTCs in the blood circulation. Their half-life was estimated to be short, ranging from 1 to 2.4 h [73]. Approximately one CTC was present in 1 billion blood cells at a given time [74], despite the fact that CTCs were constantly replenished every few hours [73], or even more CTCs entered into the bloodstream from the tumor tissues [75]. However, the detection of CTCs is still prognostic in relation to primary or non-metastatic cancer, so it has been used in current clinical trials with varying degrees of success [76,77]. Besides, CTC count was identified to be an independent predictor of PFS and OS in patients with metastatic cancers [69,70,71]. In metastatic cancers, such as prostate, pancreatic, colon, lung, and breast cancers, a higher level of CTCs with 5–5000 CTCs per mL of the blood was detected compared with non-metastatic cancers [78,79,80,81], suggesting the possible relevance of CTCs to the occurrence of metastasis.

A recent study analyzed 16 blood samples from localized HCC patients, and suggested that CTCs exhibited dynamic changes with epithelial and mesenchymal composition when they were released from the primary tumor to the peripheral vein. CTCs entered into the circulatory system with epithelial phenotypes (e.g., EpCAM, cytokeratin (CK) 8/18/19), and then dynamically switched its cellular identity to mesenchymal cells (e.g., twist, vimentin) in the circulation. The acquisition of mesenchymal characteristics of CTCs took place in the bloodstream but not in the carcinoma in situ. Interestingly, in comparison with venous vessels, the change was more likely to happen when CTCs flowed through the arterial vessels [82]. Under this circumstance, interfering EMT of CTCs in the peripheral circulation might inhibit their metastatic abilities, which in turn influences the therapeutic outcomes of the patients. Therefore, we proposed that CTCs are the precursors of HCC metastasis.

Metastasis is the main target to fight against cancer-related deaths. In the past decade, numerous studies have focused on studying the intrinsic and extrinsic mechanisms of cancer in relation to metastatic behaviors [83]. As CTCs were demonstrated to play a pivotal role in tumor metastasis, the use of CTCs in metastasis-related tumor study might shed light on cancer management. The assumption is that CTCs are the precursors of cancer metastasis, and experimental studies have also demonstrated their capability to form metastases. In addition, a study identified the function of metastasis-initiating cells in patients with breast cancer. The CTCs of patients were transplanted into the femoral medullar cavity of immune-compromised recipient mice, and it was found that liver, lung, and bone metastases were developed, which displayed similar histopathology as the corresponding patient specimen [81]. Similarly, another study reported that CTCs of patients with small-cell lung cancer were transplanted to mice, and resulted in the formation of tumors that resembled the tumor characteristics of the donor subject [84]. In HCC patients, CTCs exhibited spatial heterogeneity and displayed variation in phenotypic features. Their phenotypes were predominantly epithelial when they were released from the tumor, but were shifted to mesenchymal characteristics during travelling [82]. This process is influenced by a plethora of intra- and extra-factors, such as immunity, resistance, anoikis, and shear stresses from bloody, pleural, and peritoneal fluids [49,66]. Previous experimental data, in conjunction with the current clinical results, supported the paradigm that CTCs are the “vector” of metastasis, and they increase the metastatic propensity en route to a foreign site. Therefore, the successful capture and analysis of CTCs could act as a surrogate for characterizing the nature of the primary tumor, and provide unique insight into the metastatic process.

The metastatic process is extremely complex and is still not clearly understood. EMT has been proposed as a critical mechanism for cancer metastasis, and is involved in the pathogenesis of cancer and other human diseases [85,86,87]. It refers to a multi-step process by which epithelial cells change their differentiated phenotypes to mesenchymal stem cell phenotypes with increased cell mobility [85,88]. For the dedicated conversion between epithelial and mesenchymal states, EMT orchestrates the active mobilization of tumor cells with enhanced plasticity and migratory properties [87,89], therefore, EMT is recognized as a hallmark of tumor metastases. However, some scientists considered that EMT is not mandatory for metastasis, and alternative models have been used to study metastasis, including CTCs, which are detached from the tumor, invade into the blood circulation, and activate metastasis [82,90,91]. With the fact that CTCs enter into the circulatory system with epithelial phenotypes and dynamically switch to mesenchymal phenotypes during circulation, it is theoretically possible that CTCs are highly relevant to EMT.

In fact, the metastatic process is highly inefficient, as only a very small subset of CTCs can be capable of initiating metastasis in the distant sites [69,70,71]. Besides, tumor cells in the blood circulation are heterogenous, so simply counting the number of CTCs for clinical management is seriously in doubt. Therefore, identifying the metastatic CTCs, that are truly representative of the primary tumor, can provide valuable information to practical tumor evaluation. In the past decades, the function of CTCs in the process of cancer metastasis has been under investigation [13], and a number of specific proteins with EMT features have also been characterized [92,93,94,95]. Practically, most of the HCC patients could not be diagnosed until abnormal symptoms of metastatic lesions were developed, therefore monitoring CTCs at an earlier stage before they metastasize appears to be a potential method for HCC detection.

## 4. Potential Biomarkers of CTCs in HCC

With the complexity of the human microenvironment, CTCs are challenged by a plethora of environmental stresses, including blood components and other environmental factors. As there are some debates about CTCs, in this section, we discuss the controversial and basic questions in relation to CTCs. We also highlight the potential biomarkers of CTCs in HCC patients (Table 1), which may help to categorize potential targets for therapeutic strategies, and contribute to the widespread application of CTCs in HCC management.

### 4.1. Is CTCs Heterogeneity Compatible with EMT Markers?

The clinical significance of CTCs in HCC management has not been comprehensively discussed in other literature. We proposed that EMT-related molecules might serve as a promising indicators for monitoring CTC movement in patients with HCC, and cost-effective biomarkers for the observation of tumor progression. In this section, we critically review the clinical parameters and summarize the specific protein characteristics of CTCs that are associated with EMT.

EpCAM is one of the most common epithelial-specific cell-surface markers, and it has been widely used to isolate CTCs in experiments [96]. CTCs expressing EpCAM were detected in 120 control subjects and 299 patients with HCC who underwent resection, TACE (transcatheter arterial chemoembolization), or radiotherapy [16]. Patients with pre-operative detectable CTCs had a significantly higher recurrence rate or worse PFS than those without detectable CTCs. Moreover, the dynamic changes of CTCs were also investigated during the perioperative period. The number of EpCAM^+^ CTCs was decreased significantly after operation, and all the patients with CTC reduction showed tumor remission [16]. Similarly, EpCAM-positive CTCs were consecutively analyzed in a cohort of 78 patients. Among them, 59 patients were diagnosed with HCC and 19 patients were control subjects [17,97]. A total of 18 out of 59 (30.5%) patients had more than one CTC per 7.5 mL of blood, whereas only 1/19 (5.3%) patients without HCC tested positive with one CTC per 7.5 mL of blood. Subsequent test revealed that OS was significantly decreased in HCC patients with EpCAM^+^ CTCs compared to HCC patients without CTCs. Therefore, CTCs in the presence of EpCAM are strongly correlated to tumor aggressiveness, and this marker allows adequate stratification of HCC patients for curative or systemic therapy.

EMT is associated with a range of molecules, which are generally classified into epithelial and mesenchymal phenotypes based on their expression patterns in the process of EMT. The epithelial biomarkers include E-cadherin, CK 8/18/19, and EpCAM, whereas mesenchymal markers constitute N-cadherin, vimentin, as well as a variety of transcription factors such as twist, snail1, and slug [85,87,98,99]. In the past, only epithelial markers were used to isolate CTCs, however recent studies showed a strong association of mesenchymal CTCs with tumor progression in HCC patients. The mesenchymal CTCs were most likely to be present in advanced stage patients, and were associated with decreased relapse-free survival. In contrast, there was no difference in epithelial CTC counts between patients with and without tumor recurrence [19]. In addition, a prospective study was conducted in 62 HCC patients who underwent radical resection, and suggested that positive peripheral mesenchymal CTCs had a higher risk of getting early tumor recurrence [23]. Twist^+^ CTCs were detected in 54 out of the 80 (67.5%) HCC patients [18], the ratio of twist^+^ CTCs was positively correlated with some clinical parameters, including portal vein tumor thrombi, tumor-node-metastasis (TNM) staging, alpha-fetoprotein (AFP), cirrhosis, tumor number, tumor size, and microvascular invasion. Meanwhile, the 1-year follow up period in 33 HCC patients who underwent hepatectomy showed that the ratio of twist^+^ CTCs was closely correlated with the rate of metastasis or recurrence and the mortality rate. The ROC (receiver operating characteristic) curve analysis also suggested that the prognostic evaluation of twist^+^ CTCs was better than twist^−^ CTCs. In another study, twist and vimentin expressions were detected in CTCs from 39 (84.8%) and 37 (80.4%) out of the 46 patients, respectively. Co-expression of twist and vimentin in CTCs was detected in 32 (69.6%) out of the 46 patients, and was significantly correlated with portal vein tumor thrombi, TNM classification, and tumor size. Therefore, the levels of twist and vimentin in CTCs could serve as promising biomarkers for evaluating metastasis and prognosis in HCC patients [22].

CTCs may undergo a dynamic process of EMT that could lead to up- and downregulation of epithelial and mesenchymal phenotypes, and CTCs that have passed through a partial or complete EMT are undetectable [63]. Therefore, it is likely that the use of the combination of epithelial and mesenchymal markers could represent an alternative strategy, which is better than using a single marker, thereby improving the sensitivity and accuracy of CTC detection [100]. According to the EMT phenotypes, CTCs are classified into different subtypes, epithelial CTCs, mesenchymal CTCs, and mixed/hybrid (epithelial and mesenchymal) CTCs [24,25]. The most common method to isolate CTCs is the immunoaffinity-based CTC enrichment technique. This technique uses antibodies against CD45 to isolate and remove contaminated leukocytes from the samples, followed by the capture of targeted CTCs by using specific antibodies to detect tumor-associated antigens which are expressed on the surface of CTCs [64]. In fact, hybrid CTCs were identified in a variety of cancers. For example, the epithelial and mesenchymal markers were co-expressed in CTCs from all the patients with non-small lung cancer [94], and similar observations were seen in 84% of CTCs in prostate cancer patients and 75% of CTCs from patients with breast cancer [95]. Similarly, accumulating findings demonstrated the importance of mesenchymal and hybrid CTCs in patients with HCC. In a cohort study with 57 patients with non-malignant liver diseases and 113 HCC patients, the number of CTCs with all the phenotypes was significantly higher in the peripheral blood of HCC patients. Further analysis showed that the total number of CTCs was more effective than AFP for the diagnosis of HCC, and the combination of total CTCs and AFP could enhance the diagnostic effectiveness [20]. Moreover, the EMT phenotypes of isolated CTCs were analyzed in 195 HCC patients, the percentages of patients with epithelial, mesenchymal, and hybrid CTCs were 53%, 57%, and 83%, respectively. CTCs expressing mesenchymal markers, which are mesenchymal and hybrid CTCs, were shown to have better invasive and metastatic abilities than epithelial CTCs. Besides, a study showed that 16 patients with tumor recurrence had a higher proportion of mesenchymal and hybrid CTCs [21], and similar results were observed in another study with 33 HCC patients [24].

A recent study was conducted in 112 HCC patients, and demonstrated that BCAT1 was upregulated in CTCs from 79 patients, which is one of the cancer-related genes in HCC. The detailed analysis showed that as opposed to epithelial CTCs, more mesenchymal and hybrid CTCs tested positive for BCAT1. Given that patients with a higher percentage of mesenchymal CTCs had significantly shorter time of tumor recurrence, therefore, the effect of BCAT1 was also examined in the EMT process. Unexpectedly, the percentage of BCAT1 was positively correlated with the EMT process, suggesting a potential marker for CTCs to evaluate tumor metastasis and recurrence [26].

Taken together, CTCs are partially involved in the EMT process, and its heterogeneity is to an extent associated with EMT markers. Therefore, this section provides an alternative method of tumor evaluation, especially in patients with advanced disease.

### 4.2. Hepatocyte-Specific Markers of CTCs in HCC 

Notably, not all the CTCs are prone to entering the EMT process simultaneously, and only a few can initiate metastasis at one time [101], so EMT-based identification of CTCs limits their clinical application in HCC. Elevated levels of AFP were known to be correlated with an increased tumor size and portal vein thrombi, as well as increased risks of liver transplant waitlist dropout and post-transplant recurrence [102]. Serum AFP is a predictor of treatment responsiveness in HCC patients after liver transplant and ramucirumab treatment [103]. It is also the main HCC screening biomarker recommended by clinical practice guidance in the field of cancer [104,105,106]. However, the detection rate of AFP in the early stage of HCC is only 25–65%, this causes a big challenge for the early detection and therapy of HCC, especially with false negative results in most of the cases [107]. Recently, a novel prognostic factor, apolipoprotein A1 (ApoA-1), was identified to be correlated with CTC levels. In patients with a higher number of CTCs, those with a lower ApoA-1 level had higher recurrence rate and shorter survival time [108]. Moreover, biomarker discovery and analysis is now focusing more on CTCs with specific phenotypes. CTCs from patients tested positive for EpCAM, epithelial membrane antigen (EMA), CK18, AFP, glypican-3 (GPC3), and CK [36]. Therefore, the identification of proteins, that are exclusively located on the surface of CTCs, can overcome the challenge of low detection rate. Besides, a range of specific proteins on the surface of CTCs, including GPC3, asialoglycoprotein receptor (ASGPR), and hepatocyte paraffin 1 (Hep Par 1), was shown to provide diagnostic and prognostic information on HCC progression for its early detection and prognostication, as well as prediction and monitoring for treatment responsiveness.

GPC3, an oncofetal protein that is expressed on the cell surface of CTCs, was significantly upregulated in the early stage of HCC compared to AFP expression, and 43 out of the 68 (63.2%) AFP-negative patients had elevated GPC3 levels [27]. GPC3 mRNA was detected in 74.8% of HCC patients, while it was only detected in only 3.2% of normal liver [109]. Since then, accumulating evidence demonstrated that elevated GPC3 expression was detected in patients with HCC at both protein and mRNA levels [110,111,112,113]. The studies linked GPC3 to AFP and further characterized their diagnostic value. It was found out that this could significantly improve diagnostic efficacy [27,28]. Among 85 HCC patients, GPC3-positive CTCs in tumors were detected with a sensitivity of 60%. Therefore, both GPC3-positive CTCs and high AFP level could be predictors for microscopic portal vein invasion, and detection of GPC3-positive CTCs was better than that of AFP in terms of specificity and sensitivity [28].

ASGPR is a receptor that is specific to hepatocytes, and it is exclusively localized on the outer membrane of liver-derived cells, including hepatocytes and HCC cells [29,30,114]. CTCs in the presence of ASGPR were detected in all examined 36 and 16 patients with HCC of two individual studies, whereas ASGPR failed to be detected in extrahepatic tissues, benign liver specimens, and non-HCC cancer subjects [29,30]. Pang et al. proposed a “golden standard” for HCC detection, they combined antibodies against ASGPR and GPC3 for the detection of CTCs to ensure that no target cells were missed, and the dual labelling of CTCs could be detected with high specificity in all eight blood samples from HCC patients. Besides, no detectable CTCs in the presence of ASGPR and GPC3 were found in breast cancer patients and healthy subjects [31]. Another study enrolled 62 HCC patients and seven chronic HBV-infected patients, and similar results showed that higher expressions of GPC3, ASGPR, and CK8/18/19 were detected in the blood of HCC patients, but not in HBV-infected and healthy individuals [32].

As the big challenge of high heterogeneity in HCC cells, the incorporation of several specific markers is more likely to be effective for clinical use. Wan et al. developed a panel containing three HCC markers, GPC3, Hep Par 1, and glutamine synthase (GS) [33]. Hep Par 1 is believed to be a component of the membrane of the hepatocellular mitochondria, and does not present in the mitochondria of other normal tissues [115]. With the positive staining of Hep Par 1 in 75–90% of HCC specimens, Hep Par 1 appears to be a specific protein, which is unique to HCC [116]. Another protein GS was also observed more frequently in HCC samples than non-HCC samples [117], and its expression was shown to be increased in advanced HCC patients and was associated with shorter relapse-free survival [118]. As hypothesized, a pilot study reported that 37 out of 42 (88.1%) HCC patients tested positive for GPC3, Hep Par1, and GS in CTCs, whereas all five non-HCC subjects tested negative. Besides, CTCs were detected in 96.2% of patients with advanced HCC stage, while only 75% of patients with early stage of HCC were detected with CTCs [33].

An antibody cocktail of ASGPR and Hep Par 1 was used to monitor patients with HCC. A study reported that no CTCs were detected in healthy subjects, benign liver disease, or non-HCC cancer patients, and CTCs in presence of ASGPR and Hep Par 1 were identified in 69 out of 85 (81%) patients, with an average of 19 ± 24 CTCs per 5 mL of blood [34]. Both the positive rate and the number of CTCs were significantly correlated with the tumor size, portal vein tumor thrombi, differentiation status, and disease progression, which were classified by the TNM classification. In addition, CTC staining with combined antibodies of P-CK and carbamoyl phosphate synthetase 1 (CPS1), a newly identified antigen for Hep Par 1 [119], were detected in 24 out of 27 (89%) patients with HCC, so this provided a better CTC detection method with an average of 20% that was consistently achieved compared to single-antibody-based method [30]. The same group was then used to examine CTCs using a mixture of antibodies against ASGPR and CPS1. CTCs that tested positive for ASGPR and CPS1 were detected in 29 out of 32 (91%) patients with HCC, and there were no CTCs detected in healthy volunteers and patients with other kinds of cancers, including breast, lung, esophageal, gastric, and colorectal cancers [35].

Collectively, specific markers have been increasingly used to detect and characterize CTCs, as the sensitivity and specificity for HCC evaluation have been improved. HCC tissue is highly heterogeneous, and CTCs that were detected by a single marker might fail to provide timely and accurate information, as some CTCs that tested negative for specific markers might be missed when using the single marker-based strategy. Alternatively, a panel of several different biomarkers could be concurrently used, and it has been proved to minimize false negative or positive outcomes, as well as increasing analytical sensitivity and effectiveness.

### 4.3. How Do CTCs Respond in the Microenvironment?

In the complexity of human microenvironment, CTCs are challenged by a plethora of environmental stresses in the blood circulation [120]. Different physical factors could lead to different fates for CTCs. A few CTCs might fail to prevent from being killed in a very hostile environment, but some CTCs could survive. Aggressive CTCs that remain in the body can undergo phenotypic and functional alterations to protect themselves from environmental stresses [121,122]. In a study of small-cell lung cancer, CTCs from patients with platinum and etoposide chemotherapy were isolated and injected into the flanks of immune-compromised mice. The resultant CTC-derived explants had similar responses to chemotherapy as the donor patients, suggesting its vital role in the microenvironment [84].

A recent study identified a candidate marker, insulin like growth factor binding protein 1 (IGFBP1), in CTCs of 25 HCC patients, and this marker was shown to be correlated with the responsiveness to selective internal radiation therapy, which is a local ablative technique for HCC treatment [39]. In addition, the sensitivity of CTCs to chemotherapeutic drugs was tested by another study. When cultured with oxaliplatin or sorafenib, two representative anti-cancer drugs for HCC, CTCs from the blood samples had lower ability of forming spheroids [29]. Sorafenib is the first approved oral drug for the treatment of HCC [123]. Taken together with the data from the large randomized clinical trials, sorafenib improved the survival of HCC patients with higher efficacy and better safety [124]. However, in fact, not all the patients responded equally to sorafenib treatment [125]. Recently, Li et al. found that phosphorylated ERK (pERK) and pAkt expressions in CTCs were correlated to sorafenib efficacy in HCC patients, and pERK^+^/pAkt^−^ CTCs were the most sensitive CTCs in response to sorafenib. A sharp decline in CTC count was observed in patients with pERK^+^/pAkt^−^ CTCs after two weeks of sorafenib treatment, and CTCs from patients with a higher proportion of pERK^+^/pAkt^−^ CTCs were less likely to form spheroids. Therefore, they suggested that the population of pERK^+^/pAkt^−^ CTCs could serve as a potential predictive factor for HCC patients treated with sorafenib [37].

Liu et al. found that some CTCs interacted with adhesive myeloid-derived suppressor cells, where they created a defensive shield against immune responses and facilitated distant metastasis of malignancies [126]. Likewise, Hamilton et al. reported that CTCs recruited tumor-associated macrophages and induced metastatic behavior [127,128]. Therefore, identifying the unique and heterogeneous CTCs with the capability of escaping from immune responses might partially explain the high metastasis rate and development of HCC in the clinic. A study explored the interaction between CTCs and tumor immune microenvironment in a total of 49 HCC patients, and the prognostic value of CTCs with the combination of regulatory T cells (Treg) for post-operative recurrence was evaluated. The early recurrence rate in the group with combined higher EpCAM^+^ CTCs and Treg/CD4^+^ levels was significantly higher than that in the combined low-level group, so elevated Treg/CD4^+^ cells could cause immune suppression and contributed to CTC escape from the peripheral immune clearance. Thus, the study concluded that the combined detection of EpCAM^+^ CTCs and Treg/CD4^+^ might provide a novel prognostic predictor for HCC patients [38]. Therefore, the interplay of CTCs within the microenvironment is certainly important for tumor study, and the study of the interaction network with microenvironmental factors may help to develop potential therapeutic targets to prevent metastasis in patients with HCC.

### 4.4. Are CTCs Equivalent to Cancer Stem Cells (CSCs)?

As described above, CTCs have the ability to induce tumor progression and undergo metastasis and recurrence. Interestingly, the theory of “CSCs” shows that a minority sub-population of cells exhibit indefinite self-renewal, proliferation, and differentiation capabilities, and they are believed to be the cause of tumor initiation, progression, metastasis, and recurrence [129]. The critical role of CSCs was identified in the mouse xenograft experiments with various tumors. The stem-like cells were significantly prone to form tumors compared to non-stem-like cells [130,131,132]. Besides, CSC biomarkers were validated in some studies, such as CD133, CD13, and EpCAM, where they exist in a minor fraction with tumorigenic ability in HCC [133,134,135,136,137]. The stem-like markers, CD133 and EpCAM, were also shown to be co-expressed in HCC [138].

A prospective study used CD90 as a stemness biomarker. CD45^-^CD90^+^ cells were detected in the blood of 91.6% of HCC patients, but none were detected in normal subjects or cirrhotic patients without HCC [139]. The quantity of CD45^-^CD90^+^ in the blood circulation was positively correlated with that in the tumor tissues, and could be used to predict HCC recurrence [140]. It also indicated that cancer cells with stem-like features, that travelled through the blood circulation, were highly tumorigenic compared to those in the tissues [141]. Based on these findings, we suggest that CSCs might be a subset or derivative of CTCs.

EpCAM is widely used to detect CTCs. Many studies have indicated that EpCAM is an important stemness marker, and is of biological and clinical significance for HCC [142,143]. In this case, CTCs in the presence of EpCAM represent aggressive stem cell-like CTCs. Recently, a study analyzed the composition of CTC subtypes. In the blood of a total of 14 HCC patients, the percentage of EpCAM^+^ CTCs was only 8.03%, and post-surgical patients with EpCAM^+^ CTCs detected showed poor prognosis [144]. In fact, the level of EpCAM in CD45-depleted peripheral blood mononuclear cells of HCC patients was higher compared to healthy volunteers [40]. Patients with higher pre-operative EpCAM^+^ CTC population developed post-operative recurrence earlier than those with a lower population of EpCAM^+^ CTCs. In addition, stem cell-like phenotypes were also investigated, CSCs biomarkers, CD133 and ABCG2, were observed in the blood samples from HCC patients with positive EpCAM^+^ CTCs [40]. Another study used a mix panel of GPC3, GS, and Hep Par 1 as biomarkers for CTC isolation. Among all the 37 patients who tested positive for CTCs, the expression of CD44^+^, a common surface marker for CSCs in many cancers including HCC [145], was observed in all the stages of HCC, indicating that CTCs with these three markers had a cancer stemness phenotype [33]. Similarly, another study also showed that CD44^+^ CTC count was higher in patients with macrovascular invasion than those without invasion [33], therefore we suggested that CTCs with stem-like features are more capable of forming metastases. In fact, there is still some crossover between CSCs and CTCs, so it is difficult to distinguish between them due to the lack of a suitable model and effective methods to identify these special subpopulations. As not all the CTCs are able to form ectopic metastases, CSCs are not equivalent to CTCs [146].

Phenotypic markers used for tumor monitoring are not exclusively specific for CTCs or CSCs, so the relation between CSCs and CTCs remains controversial. It is proposed that when both CTCs and CSCs are considered simultaneously for tumor evaluation, the cell subpopulation will have more value in disease monitoring. Annexin A3 (ANXA3) is a putative stem cell marker and its upregulation was observed in a variety of tumors including HCC [147,148,149]. A study demonstrated a positive correlation between serum ANXA3 and CD133^+^ CTCs in a large cohort of 368 HCC patients, and the population of CD133^+^ CTCs was higher in patients who tested positive for ANXA3. These data supported the idea that serum ANXA3 could stimulate and maintain the stem cell-like traits of CD133^+^ CTCs to promote tumor recurrence and metastasis [42]. The diagnostic performance of ANXA3 was better than AFP [150] and combining the use of ANXA3 and AFP markers significantly improved the predictive outcome [42]. Recently, Guo et al. used four markers, EpCAM, CD90, CD133, and CK19, for constructing a CTC detection panel to detect CTCs in 50 HCC patients and 50 healthy subjects [41]. The CTC panel was then evaluated and validated in another large cohort of 906 subjects. Particularly, post-operative HCC patients with a positive CTCs panel detection had a significantly higher recurrence rate. Compared with EpCAM, the prognostic significance of CTC panel was still retained in the EpCAM^−^ subgroup [41]. Meanwhile, in contrast to the single-marker AFP, the CTC detection panel tested positive in a greater proportion of patients with HCC. It also tested positive in both AFP-negative and -positive patients with HCC [41]. This also addressed a problem, approximately 35% of patients with HCC were AFP negative [2,151], and elevated AFP concentration was also observed in non-HCC patients and patients with chronic hepatitis or cirrhosis [152,153]. Taken together, all these data suggested that the multi-markers of CTCs, which consisted of markers for both CTCs and CSCs, could be potential indicators for diagnosis, as well as for the evaluation of therapeutic responsiveness.

## 5. Conclusions and Future Perspective

Compared to the cellular components in the blood, CTCs exist in low levels. Given the fact that the liver is supplemented by the blood, CTCs are considered to be the main cause for HCC occurrence, and are also a pivotal factor for prognosis. The liquid biopsy of CTCs provides an opportunity for timely diagnosis and treatment, especially in the context of metastasis or recurrence. To date, there is no sensitive and specific method for capturing CTCs, and the only technology that was approved by the FDA is the CellSearch system, which isolates CTCs using EpCAM. However, as tumors progress with high heterogeneity, the identification of CTCs with exclusive and specific biomarkers may provide better individualized treatment for patients with HCC. This review discussed the debatable points in relation to CTCs, as well as the molecular and genetic characteristics of CTCs and their potential markers. Among these markers, there are EMT-related (epithelial/mesenchymal/hybrid markers), hepatocyte-specific, microenvironmental-related, and stem-like phenotypes, which cannot be strictly classified due to the complexity of the microenvironment and the continuum between different stages of HCC progression. However, this review provided comprehensive and favorable insight for clinical practice. Taken together, further studies are warranted to confirm the importance of identification and characterization of CTCs for personalized medicine strategies in patients with HCC.

## Figures and Tables

**Table 1:** The overview of phenotypic features of circulating tumor cells in hepatocellular carcinoma.

Phenotypic Markers	Enrichment Method	Specimen	Key Findings	Ref
Is CTCs heterogeneity compatible with EMT (epithelial to mesenchymal transition) markers?				
EpCAM	qRT-PCR-based platform	299 HCC patients and 120 control subjects	Compared with pre-operation, the population of EpCAM^+^ CTCs decreased significantly after operation, and all the patients with CTC reduction showed tumor remission.	[16]
EpCAM	CellSearch system	59 HCC patients and 19 control patients	CTCs in the presence of EpCAM were strongly correlated with tumor aggressiveness, and this allowed adequate stratification of HCC patients for curative or systemic therapy.	[17]
Twist, GPC-3	CanPatrol system	80 HCC patients and 10 healthy volunteers	The ratio of twist^+^ CTCs was closely correlated with the rate of metastasis or recurrence and the mortality rate; the prognostic evaluation of twist^+^ CTCs was better than CTCs alone.	[18]
EpCAM, CK8/18/19, and vimentin, twist	CanPatrol system	165 HCC patients	The presence of mesenchymal CTCs tended to occur in patients with advanced stage, and was associated with decreased relapse-free survival.	[19]
EpCAM, CK8/18/19, and vimentin, twist	CanPatrol system	113 HCC patients	The use of total CTCs was more effective than AFP for the diagnosis of HCC, and the combination of total CTCs and AFP could enhance diagnostic effectiveness.	[20]
EpCAM, CK8/18/19, E-cadherin, vimentin, twist, AKT2, and snail	CanPatrol system	195 HCC patients	Mesenchymal and hybrid CTCs had higher invasive and metastatic abilities than E type CTCs.	[21]
E-cadherin, vimentin, and twist	Flow cytometric analysis, and immunofluorescence staining	46 HCC patients	Co-expression of twist and vimentin in CTCs was significantly correlated with portal vein tumor thrombus, TNM classification, and tumor size.	[22]
EpCAM, CK8/18/19, and vimentin, twist	CanPatrol system	62 HCC patients	HCC patients with positive peripheral mesenchymal CTCs had a higher risk of early recurrence.	[23]
EpCAM, CK8/18/19, and vimentin, twist	CanPatrol system	33 HCC patients and 10 healthy volunteers	Epithelial-mesenchymal-mixed CTCs played an important role in EMT transition of HCC. The mixed CTCs might be a vital factor for intrahepatic metastasis, and mesenchymal CTCs could have potential to be a predictor of extrahepatic metastasis.	[24]
EpCAM, CK8/18/19, and vimentin, twist	CanPatrol system	40 HCC patients	The average ratio of mesenchymal CTCs in each sample was increased in the later stages of cancer compared with the earlier stages of cancer.	[25]
EpCAM, E-cadherin, CK8/18/19, vimentin, and twist, BCAT1	CanPatrol system	112 HCC patients	The percentage of BCAT1 was positively correlated with EMT process, suggesting a potential marker for CTCs in evaluating tumor metastasis or recurrence.	[26]
Hepatocyte-specific markers of CTCs in HCC				
GPC3, AFP	Enzyme-linked immunoassay	68 HCC patients	The combination of GPC3 and AFP improved the overall sensitivity for HCC; the positive rate of GPC3 was significantly higher than that of AFP in HCC patients.	[27]
GPC3	Density gradient centrifugation and immunomagnetic positive enrichment	85 HCC patients	Pre-operative GPC3-positive CTCs was a risk factor of microscopic portal vein invasion and poor prognosis, and therefore it might be a useful biomarker for HCC patient outcomes.	[28]
ASGPR	Microfluidic chip	36 HCC patients	CTCs were detected in all the examined patients with HCC.	[29]
ASGPR, CPS1, P-CK	Density gradient Ficoll-Paque PLUS, and magnetic labeling and separation	27 HCC patients	All the 16 HCC tissues had ASGPR staining on the membranes of the HCC cells, and CTCs in the presence of CPS1 and P-CK were detected in the majority of patients with HCC.	[30]
ASGPR, GPC3	Magnetically assisted surface-enhanced Raman scattering biosensor	Eight HCC patients, three breast cancer patients, and three healthy controls	Dual labelling of ASGPR and GPC3 was effective in detecting HCC CTCs with a small volume of blood samples in clinical settings.	[31]
ASGPR, GPC3, CK	Semiquantitative immunocytochemistry	62 HCC patients, seven HBV-infected patients, and 15 healthy individuals	The cells obtained from the blood of HCC patients had significantly higher levels of ASGPR, GPC3, and CK than cells derived from chronic HBV-infected patients or healthy controls; ASGPR, GPC3, and CK might be valuable as HCC biomarkers for CTC detection; the expression of ASGPR and GPC3 might be helpful for understanding OS of the patients.	[32]
Hep Par 1, GPC3, GS	Label-free Labyrinth technology, and immunoaffinity-based CTC-Chip (Microfluidic chip)	42 HCC patients, four non-HCC patients	The HCC CTC detection rate was improved by using three HCC markers compared to EpCAM-based identification method.	[33]
ASGPR, Hep Par 1	Magnetic separation and immunoidentification	85 HCC patients, 37 patients with benign liver diseases, 20 healthy volunteers, and 14 patients with other advanced cancers	No healthy, benign liver disease, or non-HCC cancer subjects were detected with CTCs. CTCs were identified in 69 of 85 HCC patients.	[34]
ASGPR, CPS1	Density gradient Ficoll-Paque PLUS, magnetic labeling, and separation	32 HCC patients, 17 patients with other types of cancer, 40 patients with other liver diseases, and 20 healthy volunteers	CTCs that tested positive for ASGPR and CPS1 were detected in 91% of patients with HCC, and there were no CTCs detected in healthy volunteers and in patients with any other kinds of cancers, including breast, lung, esophageal, gastric, and colorectal cancer.	[35]
CK, EpCAM, EMA, CK18, AFP, GPC-3, and Hep Par 1	BenchMark XT Slide Preparation system	23 HCC patients, six patients with non-HCC	57.1% of patients tested positive for EpCAM, 42.9% for EMA, and 21.4% for AFP.	[36]
How do CTCs respond in tumor microenvironment?				
phosphorylated ERK (pERK) and pAkt CTC	Density gradient centrifugation, magnetic separation	109 HCC patients	Phosphorylated ERK (pERK) and pAkt expressions in CTCs were correlated to sorafenib efficacy in HCC patients; pERK^+^/pAkt^−^ CTCs were mostly responsive to sorafenib; the population of pERK^+^/pAkt^−^ CTCs could be a potential predictive factor for HCC patients treated with sorafenib.	[37]
CD4^+^CD25^+^Foxp3^+^ Treg cells	PCR and fluorescence-activated cell sorting	49 HCC patients	The early recurrence rate in the group with combined higher EpCAM^+^ CTCs and Treg/CD4^+^ population was significantly higher than in the combined lower CTCs and Treg group; the combined detection of EpCAM^+^ CTCs and Treg/CD4^+^ might provide a novel prognostic predictor for HCC patients.	[38]
IGFBP1	Density gradient centrifugation, and immunomagnetic beads	25 HCC patients	IGFBP1 was correlated with the responsiveness to selective internal radiation therapy.	[39]
Are CTCs equivalent to CSCs (Cancer Stem Cells)?				
EpCAM, CD133	CellSearch system	123 HCC patients	CSC biomarkers CD133 and ABCG2 were observed in the blood samples of HCC patients with positive EpCAM^+^ CTCs.	[40]
GPC3, GS, Hep Par 1, and CD44	Label-free Labyrinth technology, and immunoaffinity-based CTC-Chip	37 HCC patients	CTCs with the expression of CD44 were observed in all the stages of HCC; CTCs with these three markers, GPC3, GS, and Hep Par 1 had a cancer stemness phenotype.	[33]
EpCAM, CD133, CD90, CK19, ABCG2, CD44, ICAM1, CD24, and Nestin	qRT-PCR	956 HCC patients and 50 healthy donors	Compared with EpCAM, the prognostic significance of CTC panel (EpCAM, CD90, CD133, and CK19) was still retained in the EpCAM^−^ subgroup.	[41]
CD133, ANXA3	Enzyme-linked immunosorbent assay	368 HCC patients	Serum ANXA3 could stimulate and maintain the stem cell-like traits of CD133 CTCs to promote tumor recurrence and metastasis; combining ANXA3 with AFP significantly improved the outcome prediction.	[42]

## Abbreviations

AFP	alpha-fetoprotein
ANXA3	Annexin A3
ApoA-1	apolipoprotein A1
ASGPR	asialoglycoprotein receptor
CK	cytokeratin
CPS1	carbamoyl phosphate synthetase 1
CSCs	cancer stem cells
CTCs	circulating tumor cells
ctDNA	circulating tumor DNA
EMA	epithelial membrane antigen
EMT	epithelial to mesenchymal transition
EpCAM	epithelial cell adhesion molecule
FDA	the US Food and Drug Administration
GPC	glypican-3; GS: glutamine synthase
HBV	hepatitis b virus
HCC	hepatocellular carcinoma
Hep Par 1	hepatocyte paraffin-1
IGFBP1	insulin like growth factor binding protein 1
OS	overall survival
pERK	phosphorylated ERK
PFS	progression-free survival
ROC	receiver operating characteristic
RT-PCR	reverse transcriptase polymerase chain reaction
TACE	transcatheter arterial chemoembolization
TNM	tumor-node-metastasis
Treg	regulatory T cells
